# Pharmacological stimulation of the parasympathetic system – a promising means of cardioprotection in heart failure

**DOI:** 10.1038/s41440-024-01726-3

**Published:** 2024-05-21

**Authors:** Fedor Simko, Tomas Baka

**Affiliations:** 1 Institute of Pathophysiology, Faculty of Medicine, Comenius University, 81108 Bratislava, Slovak Republic; 2 3rd Department of Internal Medicine, Faculty of Medicine, Comenius University, 83305 Bratislava, Slovak Republic; 3 Institute of Experimental Endocrinology, Biomedical Research Center, Slovak Academy of Sciences, 84505 Bratislava, Slovak Republic

**Keywords:** Donepezil, Heart failure, Parasympathetic system, Treatment

Chronic heart failure (HF) has attracted the utmost attention of cardiologists for more than half a century. Insight into the pathophysiology of this tricky condition has undergone principal changes during the last decades that determined adjustments to the therapeutic approaches [1]. During the 1950s, fluid retention was considered the dominant alteration in HF. As a result of blood flow reduction to- and impaired venous return from the kidneys, both leading to renal dysfunction with fluid excess, a cardiorenal model of HF was established and diuretics and digoxin became the principal treatment modalities. Twenty years later, blood redistribution in terms of vasoconstriction of the arterial and venous system to favour blood supply to the vital tissues was presumed to participate in a circulatory imbalance, leading to the establishment of the cardiocirculatory model of HF. Vasodilators were considered a rational strategy to attenuate the increased preload and afterload of the heart and to improve peripheral perfusion. In the 1990s, clinical studies revealed that increased plasma levels of stress hormones, such as noradrenaline or renin, in HF patients were associated with accelerated mortality. These surprising findings resulted in the concept of a neurohumoral model of HF. It was based on the idea that increased catecholamine, angiotensin II (Ang II) or aldosterone production, although hemodynamically beneficial during acute heart damage, are potentially deleterious in chronic HF as a result of the stress hormones‘ proliferative, vasoconstrictive and energy-consuming actions, which outperform their hemodynamic benefit. The unfavourable impact of the stress humoral players from a long-term perspective resulted in the introduction of angiotensin/aldosterone and catecholamines antagonists in HF treatment [1]. Later, this view was expanded to include the potential benefits of counterregulatory humoral molecules with antiproliferative and vasodilative effects, such as natriuretic peptides, and the sodium-glucose cotransporter 2 (SGLT2) inhibitors with a yet unknown mechanism of cardioprotection in HF [2].

The introduction of angiotensin-converting enzyme inhibitors (ACEI) and angiotensin II type 1 receptor blockers (ARB) initiated the era of neurohumoral modulators in HF treatment with a mortality reduction of about 20%. Interestingly, the adoption of betablockers (BB) was associated with significant concerns. Several decades ago, these drugs were strictly forbidden in HF patients due to their negative inotropic effect, with the threat of pulmonary edema development. These fears were overcome with the so-called “start low-go slow” approach, which enabled the possibility for the safe management of the failing heart with the nonselective β1/β2/α1 blocker carvedilol and the β1 selective blockers bisoprolol and metoprolol, yielding more than 30% mortality reduction when added to ACEI treatment. Recently, a rapid sequencing approach of starting HF treatment suggested BB along with SGLT2 inhibitors as the first step of therapy [2].

While the anti-remodelling action of renin-angiotensin-aldosterone system (RAAS) inhibitors is generally accepted as the decisive pathomechanism of cardiac protection, the underlying pathogenesis of the benefit from BB on the failing heart is still not completely understood [3]. The BB´s favourable action on energy metabolism seems to be the dominant mechanism of their cardioprotection. However, the heart rate-reducing action of BB is probably not the principal factor of their benefit, since ivabradine, the I_f_ current inhibitor in the sinoatrial node, which selectively reduces the heart rate, decreased only the combined endpoint of morbidity and mortality in the SHIFT study without an effect on mortality in this and other trials with HF [4]. Adrenergic stimulation, besides its hemodynamic actions, exerts potentially deleterious effects on several levels [3]. The interference of catecholamines with the β-receptor - adenyl cyclase - cAMP - protein kinase A pathway or with the α1 receptor - phospholipase C - diacylglycerol/inositol triphosphate route leads to the phosphorylation of cardiomyocyte proteins activating calcium cycling [5]. These mechanisms not only underlie the enhancement of inotropy but also the elevated risk of coronary artery constriction and mitochondrial damage via calcium overload associated with a reduction of energy metabolism enzyme activity. Moreover, the conversion of catecholamines to aminochromes enhances oxidative burden, thus additionally deteriorating mitochondrial energy production. Furthermore, the uncoupling of oxidative phosphorylation by the toxic products of lipolysis and intracellular hypomagnesemia may contribute to reduced ATP formation under the catecholamine surge [5]. In addition, the sympathetic system can participate in cardiac remodelling supposedly via stimulation of pro-proliferative hormone release. Adrenergic receptor blockade may exert beneficial effects in several of these conditions in a concerted manner. The reversion of beta-receptor alterations, which are numerically and functionally downregulated within the failing heart, along with restoration of the baroreflex control might also contribute to BB cardioprotection [3].

While the clinical benefits of adrenergic system inhibition are well documented, data on the parasympathetic system modification in HF are rather sparse. The cholinergic system seems to be even more complex and less elucidated than the sympathetic one [6]. Four efferent parasympathetic nerves, including the vagus nerve innervating the heart, originate from the parasympathetic centres localised in the middle part of medulla oblongata, such as the nucleus ambiguous, nucleus tractus solitarius or dorsal motoric nuclei. The cholinergic system in the heart consists of preganglionic and postganglionic parasympathetic neurons and parasympathetic intracardial ganglions. Acetylcholine is the humoral mediator of the parasympathetic nervous system, while its activity is terminated by acetylcholinesterase-induced splitting. Acetylcholine acts on muscarinic and nicotinic receptors. Cholinergic innervation of the heart is predominantly localised in the heart atria, particularly the sinoatrial node. Thus, the bradycardic effect has been considered to be the dominant action of parasympathetic system activation, while the effects of acetylcholine on ventricular contractility, metabolism or on other humoral systems seems to be much less important. Furthermore, besides the direct effect on the heart, the parasympathetic nerves, via muscarinic receptors, can inhibit noradrenaline release from the presynaptic adrenergic nerve endings, thus reducing the sympathetic action. Moreover, the variability in muscarinic receptors underlies various cholinergic effects. In addition, the impact of cholinergic system stimulation is modified by the extent of the sympathetic system activation. Thus, the sympathetic and parasympathetic nervous systems are not just opposite players but are rather actors with complex interactions, and the net effect of therapeutic activation of the cholinergic system is a challenging topic for research [6].

**Fig. 1:**
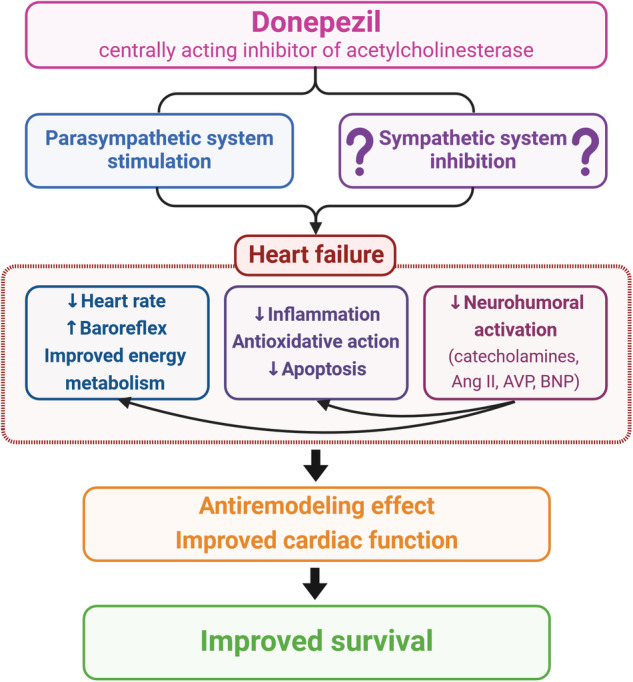
Potential mechanisms of cardiovascular benefit in heart failure by donepezil. Ang II angiotensin II; BNP brain natriuretic peptide; AVP arginine vasopressin. Created with BioRender.com

A paper published by Li and colleagues in 2004 [7] investigated rats with HF after myocardial infarction, which underwent right vagal nerve stimulation for six weeks. The intervention not only prevented cardiac remodelling and dysfunction but significantly improved the survival rate. Ten years later, the authors demonstrated the analogical functional, morphological, and survival benefit on the same model of HF by oral administration of the acetylcholinesterase inhibitor donepezil [8]. These cardiovascular benefits were later confirmed in a study with donepezil applied via an intracerebroventricular infusion [9]. In their recent work [10] published in *Hypertension Research*, donepezil suppressed the progression of cardiac remodelling and dysfunction as well as coronary artery remodelling, attenuated systemic inflammation, and reduced the plasma levels of catecholamine, angiotensin II, arginine vasopressin and B-type natriuretic peptides in a model of HF combining spontaneously hypertensive rats (SHR) with myocardial infarction. Most importantly, the 50-day mortality was impressively reduced. Mechanisms behind cardioprotection with donepezil in this and previous works may involve several levels, such as donepezil-induced reduction of systemic inflammation via peripheral α7-nicotinic acetylcholine receptor, oxidative burden reduction or attenuation of neurohumoral activation (Fig. 1). However, the causality of the particular protections in relation to the pathophysiology of donepezil benefit should be interpreted with caution, since, as the authors admit, the attenuation of the neurohumoral stimulation may not only be a direct effect of donepezil itself but may also reflect the improvement of the HF [10].

While the results of experiments with the parasympathetic system stimulation are encouraging, a number of questions for future research are emerging:

- Are parasympathetic activators equally or more effective than BB and in which indication should they be considered? Hypothetically, since donepezil seems to be well tolerated in clinical trials with Alzheimer´s disease, it could be considered an alternative to BB, in cases when BB is poorly tolerated or contraindicated. The combination of donepezil with BB may also be of benefit [11]. However, one should bear in mind the combined treatment with RAAS inhibitors, where the simultaneous administration of ACEI and ARB brought only restricted benefit and accelerated side effects. On the other hand, the addition of aldosterone blockers to angiotensin II antagonists yielded unexpected benefit [12].
- Will it be possible to consider donepezil or some other parasympathetic stimulator in the treatment of hypertension and hypertensive heart [13]?
- It would be exciting to consider whether donepezil exerts an additional benefit if added on top of currently recommended HF treatment, including SGLT2 inhibition, as there is restricted space for further improvement.
- Currently, only SGLT2 inhibitors are recommended as a treatment of HF with preserved ejection fraction [14]. Donepezil, with its anti-remodelling, antioxidant, and anti-inflammatory action, could be investigated in this indication.

A positive answer to some of these questions in a prospective large clinical trial may open a new avenue of HF treatment with parasympathetic stimulation.
